# *Escherichia coli* ST117: exploring the zoonotic hypothesis

**DOI:** 10.1128/spectrum.00466-24

**Published:** 2024-09-06

**Authors:** A. B. S. Saidenberg, S. M. Edslev, S. Hallstrøm, A. Rasmussen, D. E. Park, M. Aziz, B. dos Santos Queiroz, A. A. S. Baptista, F. Barbosa, V. G. P. Rocha, Arnoud H. M. van Vliet, A. Dalsgaard, L. B. Price, T. Knöbl, M. Stegger

**Affiliations:** 1 Department of Bacteria, Parasites and Fungi, Statens Serum Institut, Copenhagen, Denmark; 2 School of Veterinary Medicine and Animal Science, São Paulo, Brazil; 3 Section for Food Safety and Zoonoses, Institute for Veterinary and Companion Animal Science, Københavns Universitet, Copenhagen, Denmark; 4 Antibiotic Resistance Action Center, Department of Environmental and Occupational Health, Milken Institute School of Public Health, George Washington University, Washington, DC, USA; 5 Preventive Veterinary Medicine Department, State University of Londrina, Parana, Brazil; 6 Department of Comparative Biomedical Sciences, School of Veterinary Medicine, Faculty of Health and Medical Sciences, University of Surrey, Guildford, United Kingdom; 7 Antimicrobial Resistance and Infectious Diseases Laboratory, Harry Butler Institute, Murdoch University, Perth, Australia; The University of Tennessee Knoxville, Knoxville, Tennessee, USA

**Keywords:** *Escherichia coli*, ExPEC, FZEC, poultry, ST117

## Abstract

Extraintestinal pathogenic *Escherichia coli* (ExPEC) can lead to severe infections, with additional risks of increasing antimicrobial resistance rates. Genotypic similarities between ExPEC and avian pathogenic *E. coli* (APEC) support a possible role for a poultry meat reservoir in human disease. Some genomic studies have been done on the ST117 lineage which contaminates poultry meat, carries multidrug resistance, can be found in the human intestinal microbiota, and causes human extraintestinal disease. This study analyzed the genomes of 61 *E. coli* from Brazilian poultry outbreaks focusing on ST117, to further define its possible zoonotic characteristics by genotypic and phylogenomic analyses, along with 1,699 worldwide ST117 isolates originating from human, animal, and environment sources. A predominance of ST117 was detected in the Brazilian isolates (*n* = 20/61) frequently carrying resistance to critical antibiotics (>86%) linked to IncFII, IncI1, or IncX4 replicons. High similarities were found between IncX4 from Brazilian outbreaks and those from *E. coli* recovered from imported Brazilian poultry meat and human clinical cases. The ST117 phylogeny showed non-specificity according to host and continent and an AMR index score indicated the highest resistance in Asia and South America, with the latter statistically more resistant and overrepresented with resistance to extended-spectrum beta-lactamases (ESBL). Most ST117 human isolates were predicted to have a poultry origin (93%, 138/148). In conclusion, poultry is a likely source for zoonotic ExPEC strains, particularly the ST117 lineage which can also serve as a reservoir for resistance determinants against critical antibiotics encoded on highly transmissible plasmids.

**IMPORTANCE:**

Certain extraintestinal pathogenic *Escherichia coli* (ExPEC) are particularly important as they affect humans and animals. Lineages, such as ST117, are predominant in poultry and frequent carriers of antibiotic resistance, presenting a risk to humans handling or ingesting poultry products. We analyzed ExPEC isolates causing outbreaks in Brazilian poultry, focusing on the ST117 as the most detected lineage. Genomic comparisons with international isolates from humans and animals were performed describing the potential zoonotic profile. The Brazilian ST117 isolates carried resistance determinants against critical antibiotics, mainly on plasmids, in some cases identical to those carried by international isolates. South American ST117 isolates from all sources generally exhibit more resistance, including to critical antibiotics, and worldwide, the vast majority of human isolates belonging to this lineage have a predicted poultry origin. As the world's largest poultry exporter, Brazil has an important role in developing strategies to prevent the dissemination of multidrug-resistant zoonotic ExPEC strains.

## INTRODUCTION

Extraintestinal pathogenic *Escherichia coli* (ExPEC) remains one of the leading worldwide causes of urinary tract infections, meningitis, and sepsis in humans (1, 2). It also impacts several animal species, including pets, and livestock (3–5). Poultry is the primary livestock species affected by APEC (avian pathogenic *E. coli*) (6), a specific ExPEC pathotype linked to avian pathogenicity (7). The poultry industry faces huge losses due to outbreaks particularly affecting large producers such as Brazil, China, and the USA. In addition, there is an increasing therapeutic challenge to treat diseases caused by emerging multidrug-resistant APEC strains (8). Brazil is the world’s largest poultry meat exporter with 14 million tons of broiler meat exported to 151 countries annually (9) and APEC outbreaks challenge efforts to reduce antimicrobial use in the Brazilian poultry industry (10).

In addition to the risks associated with antimicrobial resistance (11), the clear genetic similarity between human ExPEC, poultry APEC, and some human clinical cases have been hypothesized to be of animal and/or foodborne origin (2, 12). Despite these similarities, including shared multilocus sequence typing (MLST) sequence types (STs), additional evidence is needed to confirm zoonotic transmission of these *E. coli* pathotypes (13, 14). Still, existing evidence supports the hypothesis of a poultry meat reservoir of *E. coli* that can colonize the human gastrointestinal tract and cause extraintestinal infections (12), similar to the well-established link between diarrheagenic *E. coli* strains and foodborne outbreaks (1). The foodborne zoonotic *E. coli* (FZEC) hypothesis has been investigated by various genomic analyses (14–16). A recent study performed in a remote city with no local poultry production analyzed source-associated mobile genetic elements to identify zoonotic *E. coli* lineages among human extraintestinal infections. An estimated 8% of clinical infections (urine and blood samples) were found caused by putative zoonotic ExPEC strains linked to meat origins. The ST117 was the most common *E. coli* lineage in the samples from poultry and one of the most common among the putative zoonotic infections (17).

ST117 is a relatively newly recognized important lineage of ExPEC, and hence less comparative studies have been done compared to other critical ST’s such as ST23, ST95, and ST131, despite the evidence of its growing importance in APEC poultry outbreaks globally (18–20). The ST117 lineage can contaminate poultry meat (12, 21) and carries multidrug resistance (MDR: resistance to three or more different classes of antimicrobials), including to highest priority critically important antimicrobial classes (20, 22, 23). Importantly, it has been detected in multiple hosts (6, 12, 24) and can colonize the human intestinal microbiota where it has been linked to extraintestinal disease (12, 25, 26).

This genomic study analyzed *E. coli* associated with disease outbreaks on Brazilian poultry farms with a focus on ST117, to provide further evidence of its zoonotic potential. We did phylogenetic comparative analyses of Brazilian and international isolates of human and animal origin. Our results implicate food animals as a source of human infections and thus further support the zoonotic hypothesis for the ST117 lineage.

## RESULTS

### Different lineages are involved in Brazilian APEC poultry outbreaks, with a predominance of ST117

*In silico* typing of the 61 APEC isolates from the four farms across three regions in Brazil revealed a variety of serotypes, phylogroups, *fim-H* type alleles, plasmid replicons, and STs associated with disease outbreaks at broiler and egg-laying farms (Table S1). These represented 23 different STs, including well-known lineages linked to APEC outbreaks (e.g., ST23 and ST101, *N* = 3 each), to human disease (e.g., ST131-*H*22, *N* = 2), or both (e.g., ST95, *N* = 2) (12). The largest number of isolates belonged to ST117 (*n* = 20/61). Most isolates were resistant to critically important antimicrobials, in particular the ST117 lineage (Table 1).

**TABLE 1:** Overall AMR presence among all APEC and the ST117 group of isolates sequenced in this study

Farm	Year	NI (ST117)^*a*^	% ST117	% ST117 AMR to critically important antimicrobials^*b*^	% other STs AMR to critically important antimicrobials^*b*^
Egg-laying/MG	2017	13 (3)	23	100	90
Broilers/RS	2019	10 (6)	60	100	100
Broilers/MG	2020	20 (4)	20	100	88
Broiler/PR	2020	18 (7)	39	86	55

^*a*^ NI: number of Isolates.

^*b*^ Fluoroquinolones, cephalosporins (3rd and higher generation), aminoglycosides, colistin, fosfomycin.

Genes encoding assorted virulence factors that contribute to extraintestinal disease were detected (27) including adhesion (*csg*, *fdeC, fim, lpf, pap, sfaX*, and *tsh*), invasion (*ibeA*), protectins/serum survival (*iss, kps, ompA*), iron acquisition (*fyu*, *hlyE, iro, irp, iuc, iut*), and toxins (*cdt, pic, vat*). Most isolates were classified as possessing ColV-like plasmids (Table S1). Long-read sequencing of the seven selected isolates allowed us to confirm the virulence factors carried on these plasmids, matching the criteria by Liu et al. (13) (Fig. S1).

### Plasmid analyses identify the carriage of critical AMR genes on specific replicons

Short-read sequencing revealed a variety of plasmids with the majority of the isolates featuring IncF-type plasmids (58/61) (Table S1). Seven ST117 isolates collected in this study were selected as representatives for highly antimicrobial-resistant variants (beta-lactams, colistin, and fosfomycin) and further analyzed by long-read sequencing (Oxford Nanopore Technologies, England) to identify the genomic composition of the underlying AMR markers. Among these, high-priority resistance genes were linked to IncFII, IncI1, or IncX4 replicons, while the detected virulence genes were located on separate IncFIB ColV-like types (Table S2). Two isolates (APEC 110 and APEC 180) additionally carried resistance markers against tetracyclines, aminoglycosides, and sulfonamides on the ColV-like plasmids carrying virulence markers (data not shown).

Identical plasmid replicon types were detected in isolates from different farms in the case of IncFII and IncX4, though some plasmid contents from different states differed (Table S2). These differences were displayed by the IncFII-positive isolates, where three isolates possessed the same pMLST type (F33:A-:B-), but had different AMR profiles. These isolates originated from farms in the state of Rio Grande do Sul (isolates APEC 79 and 85) and carried *bla*_CTX-M-55_ and *bla*_TEM-1_, with an isolate from the state of Minas Gerais (APEC 110) additionally carrying *fosA3* (Table S2).

Co-carriage of different plasmids harboring genes conferring resistance to critical antimicrobials was seen in one isolate (APEC 271) carrying *bla*_CTX-M-8_ and *mcr-1.1* on IncI and IncX4 replicons, respectively (Table S2).

**Fig 1:**
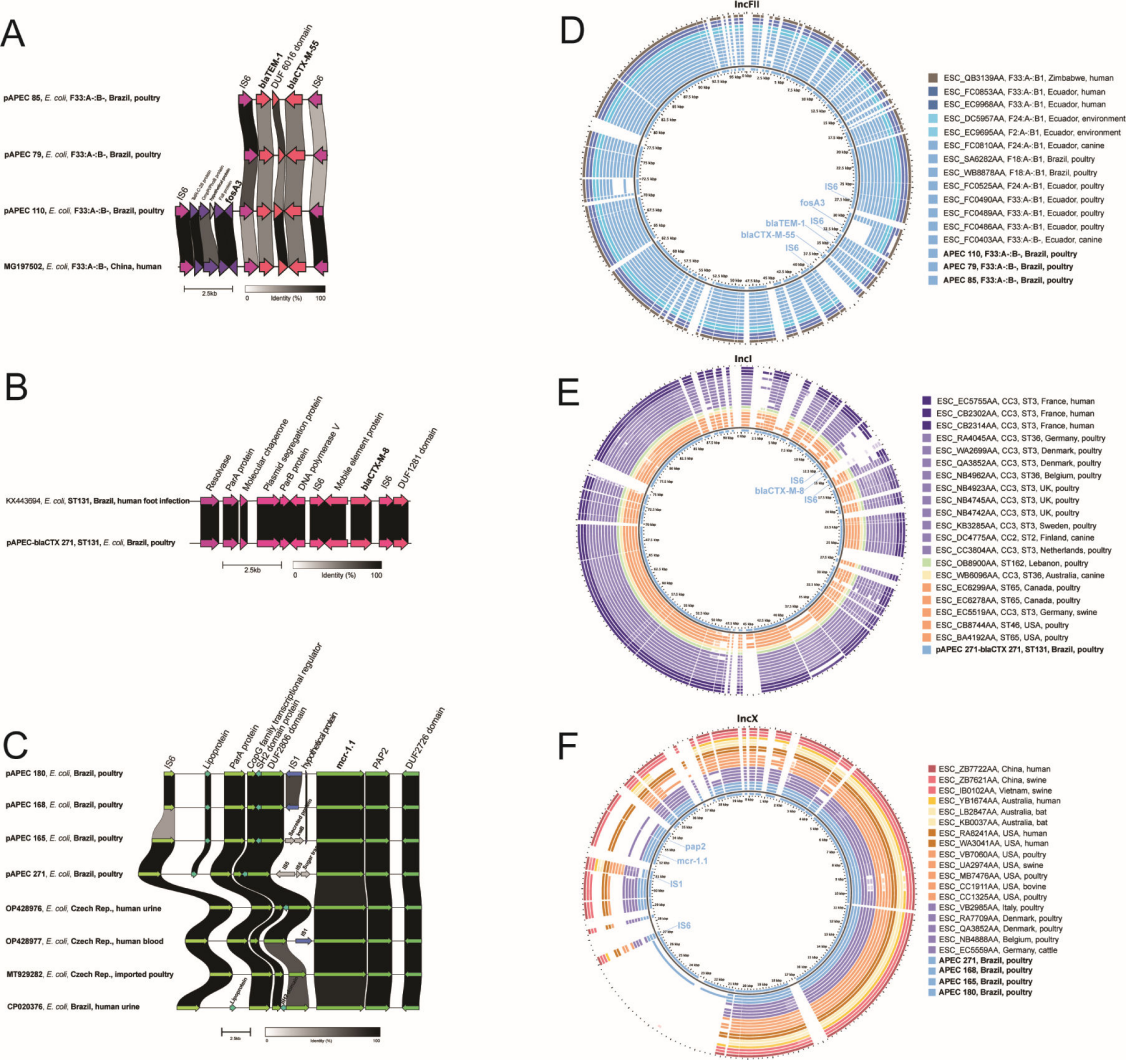
(A, B, and C) Comparison of the genetic environment surrounding high-priority AMR genes with the highest similarity (BLASTn) isolate(s). The genes of interest are in bold, and the arrows indicate the position and orientation of open reading frames. (D, E, and F): BLAST atlas comparison. The isolates of this study are highlighted in bold. The inner ring indicates one of the plasmids of this study as the reference, compared with the additional plasmids and highest identity draft ST117 sequences (outer rings). The colors represent each corresponding continent, with the host colored by different shades: darker shades identify human isolates, and lighter indicate animal isolates.

For the IncF replicon, the genetic structure surrounding the AMR genes shared conserved areas flanked by IS6 elements which were highly similar, and in one isolate (pAPEC 110) a *fosA3* gene was present adjacent to this region (Fig. 1A). Similarly, for the single IncI1 replicon detected among our isolates (pAPEC-bla_CTX_ 271), the genetic structure surrounding the *bla*_CTX-M-8_ gene also showed a similar flanking IS6 structure (Fig. 1B), while for IncX4, the genetic environment surrounding the *mcr-*1.1 gene consisted of a characteristic *mcr-pap2* cassette (28) (Fig. 1C).

### Brazilian APEC and worldwide isolates are highly variable and ST117 isolates are distributed among a variety of hosts and continents

The phylogenetic analyses of the 61 APEC isolates had SNPs called in 58% (3.78 Mb) of the core genome. There was, as expected, clustering by ST and general agreement in terms of serotypes and fimH types, with few exceptions in isolates possessing a non-typeable O antigen using the defined thresholds but lowering of threshold settings confirmed the overall clade’s main serogroup. Some fimH variation was detected in clades that shared a dominant fimH (Fig. S1). For these, only minute differences (few nucleotides) were detected among the clade’s fimH type and the divergent isolate (data not shown). Between farms, the same ST could be detected with similar AMR profiles (Fig. S2), and though for the majority of isolates, a phylogenetic link could be inferred, the core genome for most was still distinct (>100 SNPs, data not shown).

Regarding ST117 isolates, SNPs were called in 55% (2.85 Mb) of the reference genome, and fewer SNPs were noted between some subsets of isolates originating from different farms, as in the case of isolates APEC 115/127 and isolates APEC 276/280 having <21 SNPs (data not shown). In general, >100 SNP differences were observed between the Brazilian isolates from this study and the worldwide isolate collection, though there were a few exceptions that displayed fewer SNP differences (data not shown).

**Fig 2:**
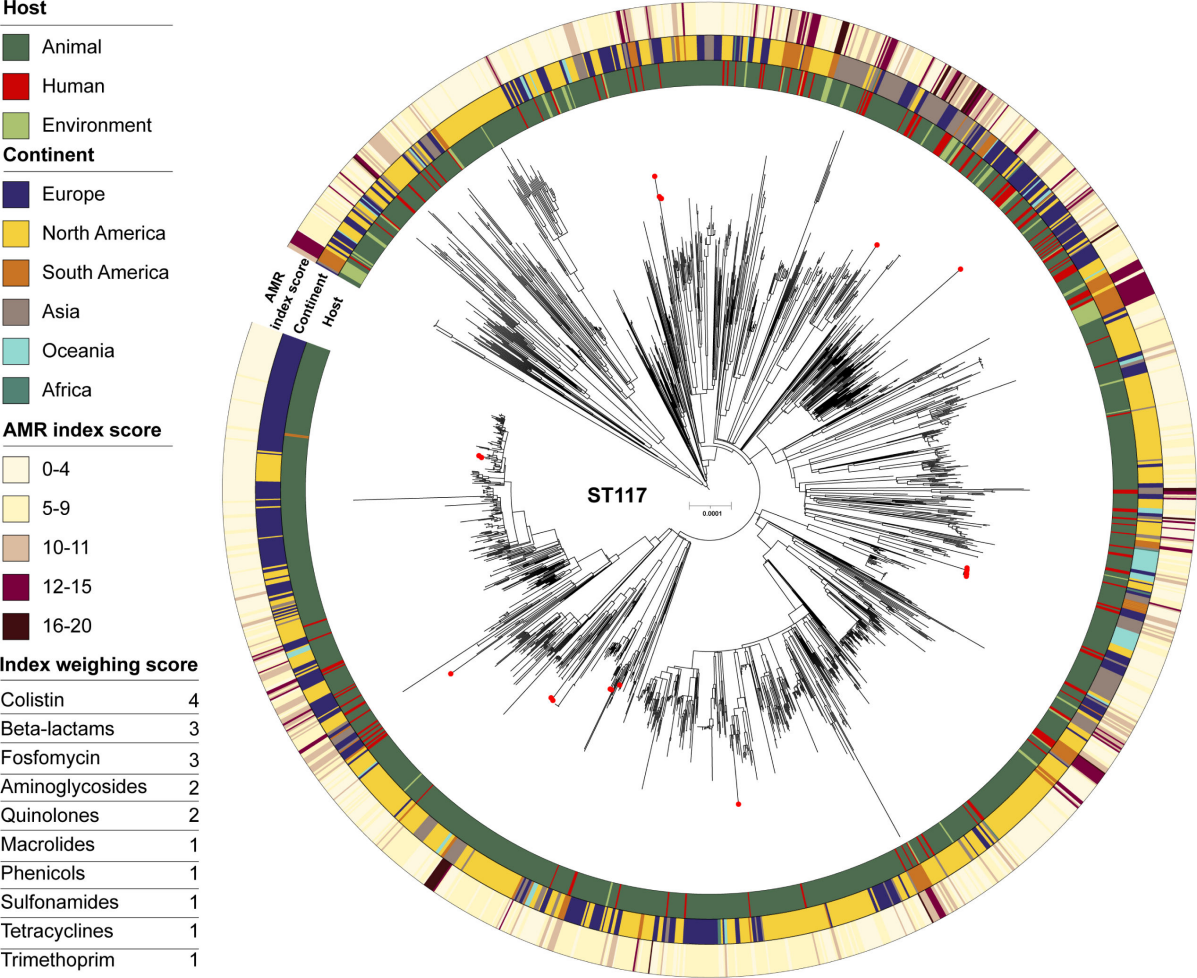
Midpoint rooted core genome phylogeny of 1,719 ST117 isolates connecting to the available metadata for host, continent, and the added overall score for each AMR class based on the index weighing score for each antibiotic class, from the inner to the outer ring, respectively. The phylogeny was built on SNP calling in 55% (2,85Mb) of the reference genome. The Brazilian poultry isolates of this study are shown with a red circle at the tip of the respective branch.

The core genome phylogeny of all 1,719 ST117 isolates (1,699 from EnteroBase) indicated no particular clustering according to source, with animal, human, and environment isolates dispersed around the tree (Fig. 2). This pattern neither indicated any predominant presence of human or animal isolates in certain continents, and no clear image linking these to the AMR indexing score by analyzing the phylogeny alone. Our Brazilian ST117 APEC isolates were distributed among isolates from various hosts and geographical origins.

### AMR scores are higher in Asia and South America

Overall, MIC results correlated with the *in silico* AMR gene predictions with exceptions related to two colistin resistance gene carriers (*mcr*-1.1) where for isolates APEC 170 and 180, the *in silico* detected resistance did not reach the breakpoint in the phenotypic test (Table S1).

**Fig 3:**
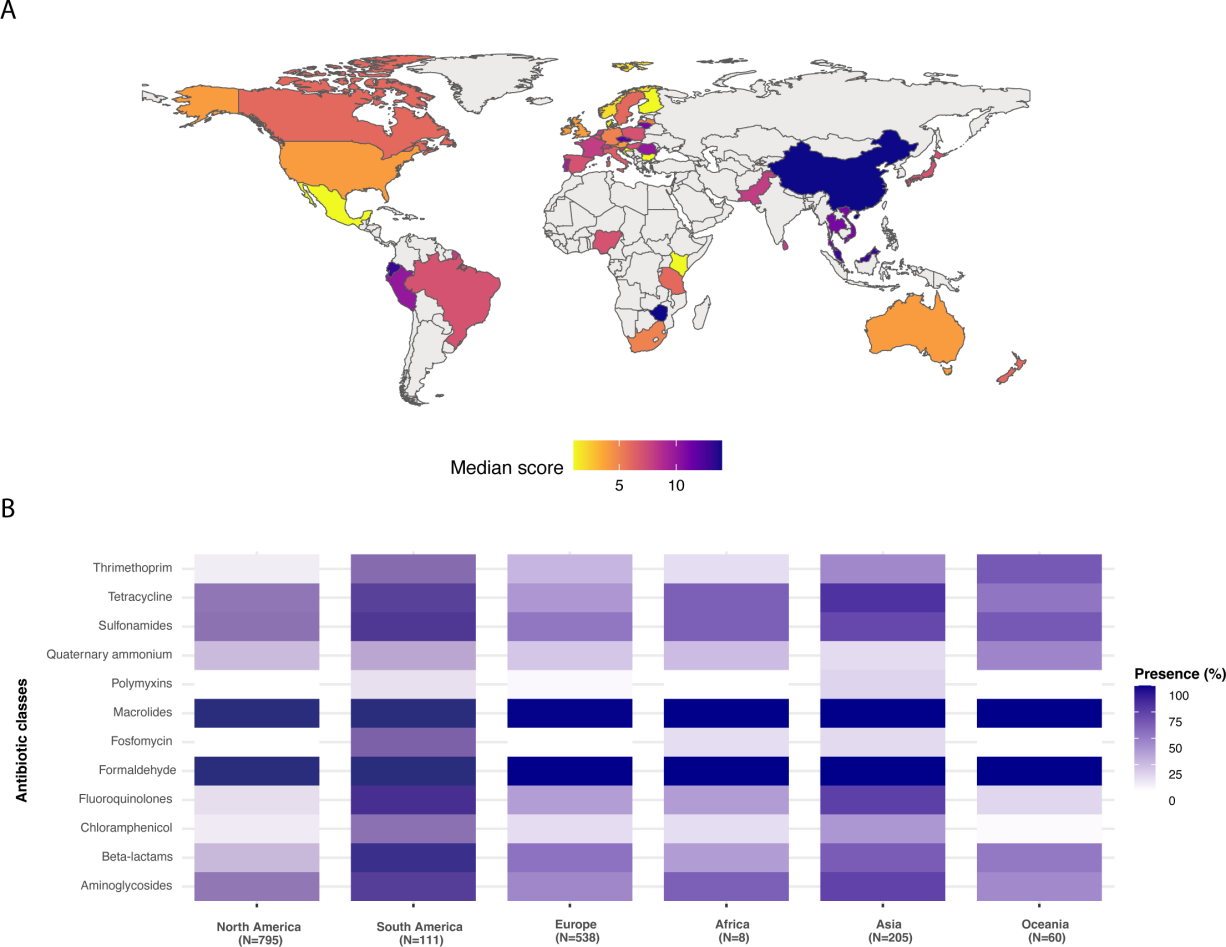
**(A)** Heat map indicating the added index weighting scores for antimicrobial classes identified in ST117 isolates according to each country. (**B) **Heat map showing the percentages of isolates from the different continents carrying genes conferring resistance to the antimicrobial class reported in the ST117 isolates. The colors in **A** and **B** indicate the lowest score/percentage (lighter) to the highest added scores (darker).

The index score highlighted several countries with high AMR scores. Although individual isolates with high scores were represented in all continents, the added highest scores were concentrated in Asian and South American countries (Fig. 3A). All of the worldwide isolates were resistant to macrolides and formaldehydes. Isolates from South America and Asia were in general more often resistant to multiple antimicrobial classes compared to other continents (Fig. 3B; Fig S3; Table S3). Some types of antimicrobial resistances were not reported in ST117 from particular continents (e.g., fosfomycin in Europe and Oceania). For Africa, fewer genomes were available for comparison analyses.

Delving into the different sources of the ST117 isolates in the global collection, it was evident that isolates of animal origin were less prone to carrying high-level AMR genes compared to isolates of human or environmental origin (Fig. S4A and S5). However, the same was not so distinctly observed for South American animal sources which more often carried AMR genes (Fig. S4B).

### Evidence for ST117 zoonotic infections

**Fig 4:**
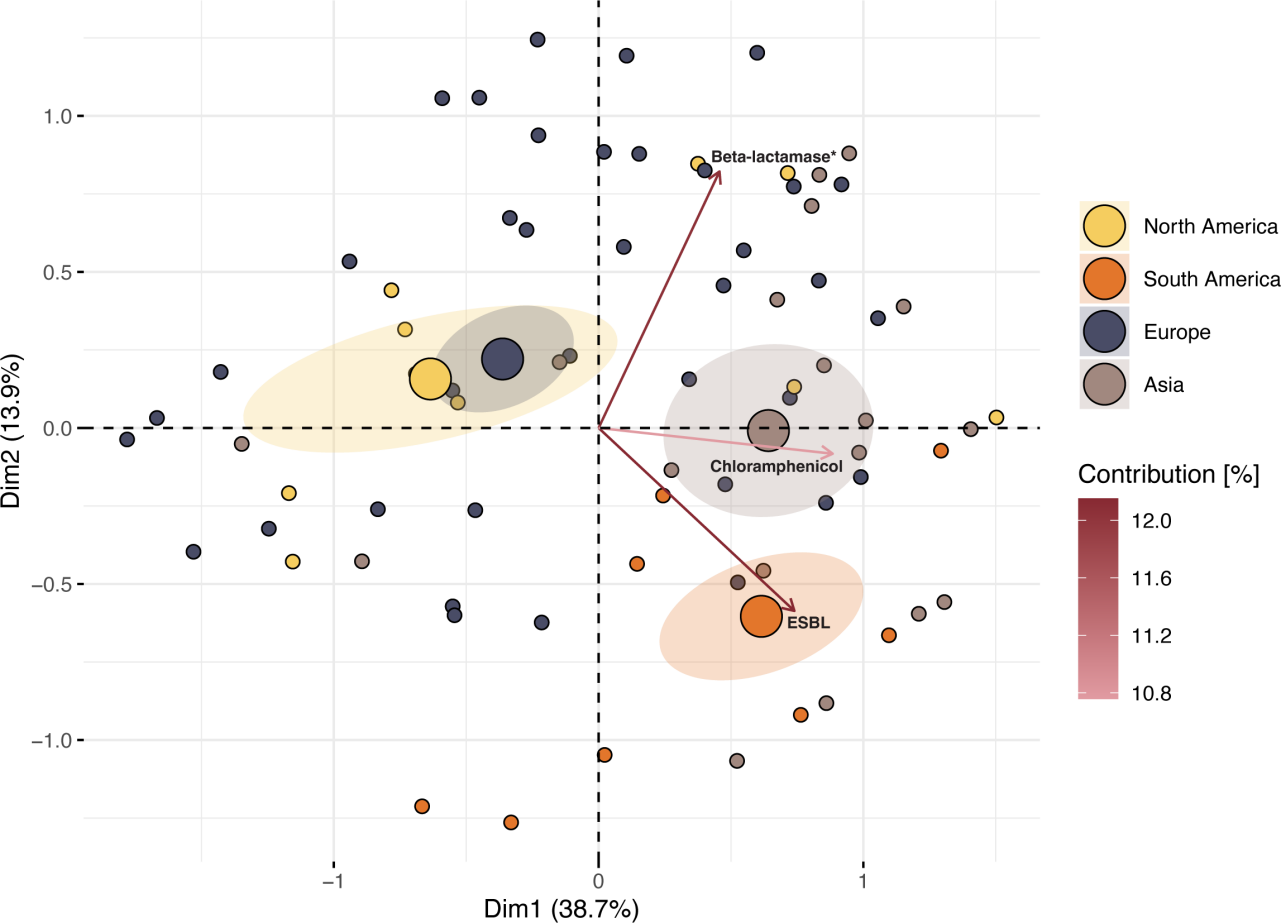
PCA biplot illustrating the clustering of putative zoonotic ST117 isolates (*N* = 126) based on the presence of resistance to antimicrobial classes. Isolates are colored based on the continent of origin. The large dots indicate the mean point within each group (continent) and the ellipses indicate the 95% confidence levels of the mean, while the arrows indicate the percentage of explained variance of each antibiotic class (top three shown). * not ESBL or carbapenemase.

The vast majority of the human isolates were predicted to have a poultry origin according to the Bayesian latent class model (93%, 138/148), with 7/10 of the non-classified being undetermined in origin, and 3/10 as human-adapted (Table S4). These three latter isolates, though different, still clustered among animal isolates in the phylogenetic analysis. Most human isolates classified as putative zoonotic carried resistance against critical antimicrobials (74.6%) (Table S4). A PCA biplot of the putative zoonotic data set indicated that North American and European zoonotic isolates share similar overall resistance patterns and that Asian and South American isolates were different in their AMR profiles and linked to specific drug classes, with ESBL being overrepresented in South American zoonotic strains (Fig. 4). As only three isolates of human origin were available from African countries, these were not included in the plot.

The plasmid comparisons of the Brazilian ST117 isolates revealed a high resemblance with isolates from multiple countries from both animal and human origins. However, they did not always share the same pMLST, and several of the international *E. coli* isolates lacked the AMR genes found in Brazilian isolates (Fig. 1A, B, and C). An IncF replicon detected in an ST117 isolate from an *E. coli* isolate from a human in China carried a nearly identical genetic region to the Brazilian poultry isolates (99.96%, Fig. 1A; Table S2). For the IncI1 replicon (pAPEC-blaCTX271), >99.9% nucleotide identity was identified with an *E. coli* plasmid from human skin infection in Brazil (Table S2; Fig. 1B). For IncX replicons, identical *mcr-*1.1 cassettes were shared with plasmids sourced from effluent water and a patient in Brazil, as well as Brazilian poultry meat exported to Czech Republic and South Korea, and with human clinical cases from the Czech Republic (Table S2; Fig. 1C).

## DISCUSSION

The investigation into outbreak-associated *E. coli* isolates from poultry in Brazil highlights that APEC clones from several genetic lineages circulate, of which some are of high importance to poultry production by causing outbreaks as well as human health giving rise to ExPEC infections. Most isolates were MDR, often to critically important antimicrobials. This was in particular the case for ST117 isolates which carried resistance genes mainly on Inc type replicon plasmids. The ST117 from this study also carried virulence factors associated with extraintestinal disease in both humans and poultry. The phylogenetic analysis with publicly available genomes revealed the presence of ST117 among human, animal, and environment sources worldwide, though more genomic data are publicly available in poultry when compared to human and environment isolates. The Bayesian model predicted poultry meat as the most probable source for the vast majority (93%) of human ST117 isolates corroborating the zoonotic origin for human cases worldwide.

For most APEC of our Brazilian isolates, there was an assortment of strains on the same farms based on their STs and core genome differences, as well as different plasmid carriage and AMR profiles, indicating their wide variability across different flocks and time periods even if processed through the same integrated farm system.

In the case of ST117, the SNP analysis did not indicate a close relationship between most of the Brazilian poultry isolates from different farms across regions even when of the same ST. However, there were exceptions where isolates originating from farms located in different states were closely related in terms of lower SNP differences, albeit with different antimicrobial susceptibility profiles, perhaps indicating farm-specific adaptation. This could be linked to the verticalization of the parent broiler production leading to a previous common supplier of 1-day-old chicks for particular batches as suggested with ST224, another dominant lineage possessing MDR found in Brazilian poultry (10), as well as ST457 in South America (29), or internationally with ST23 and ST101 (30). The information needed to trace flock origin was not available for this study.

Most of our Brazilian isolates were carriers of IncF ColV-like plasmids which indicate more virulent APEC strains (Table S1; Fig. S1). The common practice in Brazil of administering antimicrobials as prophylaxis to control disease outbreaks will put additional selection pressure, particularly on AMR genes carried on MGEs including plasmids, thus facilitating the horizontal spread of resistance genes and selecting even more challenging virulent strains (31). The long-read sequencing of selected isolates confirmed the carriage of genes conferring resistance against critical antimicrobials on a particular Inc-type plasmids (Table S2). Still, even if isolates carrying the same plasmid replicons originated from the same farm, they had mostly different AMR profiles (Table S1), a heterogeneity that has been highlighted earlier (32).

Brazilian poultry and related meat products frequently carry *E. coli* of pandemic clonal lineages resistant to critical antimicrobial classes (33). Worryingly, it is projected that the use of antimicrobials in food production will continue to increase in developing countries, and although alternatives to large-scale antimicrobial usage are currently being pursued, Brazil is already among the top five antimicrobial consumers worldwide (34). For instance, preventive use of ceftiofur in some hatcheries is still common in the Brazilian poultry sector (35), a practice that has been linked to the spread of IncF plasmids carrying different *bla*_CTX-M_ types that often co-harbor resistance genes to other critical antibiotics, in particular to fosfomycin(36) (35). The carriage of *bla*_CTX-M_ types was frequent among our isolates, particularly those belonging to ST117 (Table S1). Co-carriage of *bla*_CTX-M-55_, *bla*_TEM-1_, and *fosA3* on IncF was present in one of our isolates (APEC 110, ST117) (Table S2).

IncI plasmids carrying *bla*_CTX-M-8_ are common in Brazilian poultry (20) and were also present in two of our Brazilian isolates (Table S1). Importantly, long-read sequencing of one of these showed that it also co-carried colistin resistance (*mcr*-1.1) on IncX4 (Table S2), a profile previously reported in varied STs of Brazilian poultry meat isolates (37). An asymptomatic human case from Brazil carried an ST117 isolate harboring *bla*_CTX-M-8_ on IncI, in addition to *bla*_CTX-M-55_ and *fosA3* on IncF (26), a profile likely related to a food-animal origin. IncX4 plasmids carrying *mcr-*1.1 were present in a monophyletic cluster of four Brazilian ST117 isolates from two farms in different geographical regions where all shared similar genetic buildup surrounding the *mcr*-1.1 gene (APEC 165/168/180 and 271, see Fig. 1C) despite originating from different production systems (broilers and egg-laying) and years (2017 and 2020). This may indicate either a previous common source in higher levels of poultry production or a feed supplying source, now widespread among different farms. An IncX4 plasmid detected in an ST117 isolate from a clinical patient in the USA is highly similar to human isolates from Brazil and suggested to have an avian origin (25). The IncX4 plasmids were near-identical to plasmids found in ST117 draft sequences worldwide, of which some are not obviously linked to Brazilian poultry meat (Fig. 1F). However, highly similar IncX4 plasmids were detected in public repositories, where metadata linked their origin to poultry meat imported from Brazil as well as human clinical cases (Fig. 1C), thus emphasizing the epidemiological significance for AMR spreading through food products in Brazil, but importantly, also internationally.

Phylogenomic studies on pandemic STs with zoonotic potential show that although human or animal-only clades may be observed, the presence of clades where human and livestock isolates intermingle suggests host-transition events (13–15). However, given that these isolates tend to be widespread and that finding perfect matches is unlikely even with extensive sampling, core-genome SNP differences are not the optimal approach to infer a common origin (13). Therefore, additional methods to further evaluate the foodborne zoonotic hypothesis are needed such as investigating MGE. The ST117 lineage is strongly adapted to the avian host given its frequency in widespread poultry outbreaks (19) as well as very frequent carrier of the ColV-like type replicon (20), and poultry is overrepresented in the ST117 data set present on EnteroBase (*N* = 1,233/1,719, Table S5). However, the overall ST117 phylogeny showed no particular clades with host or country predominance (Fig. 2) confirming previous studies (5, 17, 20).

The applied Bayesian latent class model confirmed that most ST117 human isolates carry several avian-associated host markers and thus points to a classification as zoonotic (*n* = 138/148) (Table S4), indicating a link through poultry meat consumption. The few genomes of isolates not classified as zoonotic could indicate more ancestral transmission events where the isolates have lost their avian-adapted MGEs while acquiring human-associated MGEs. Further assessments are warranted as larger data sets from human sources within this lineage become available.

Pandemic ExPEC lineages are frequently adapted to the intestinal tract which acts as a reservoir for subsequent infections, without many differences between commensals and those causing disease (38, 39). This study only sampled isolates from diseased poultry in Brazilian farms, while no isolates from healthy individuals or the farm environment were compared. Therefore, additional studies on commensal and environmental isolates would be required to establish such potential zoonotic links. Among the 138 international ST117 human isolates classified as zoonotic, 22 originated from stool or perineum samples from asymptomatic individuals potentially highlighting the adaptation to persist in the human microbiota (Table S4). Interestingly, airways (including bronchi and lungs) were listed as the isolation site of four zoonotic isolates. Given that in poultry the main port of entry for APEC infections is the respiratory tract, colonization factors that facilitate the attachment to epithelial cells and then possible invasion in the human host, besides the well-characterized urinary tract infection and bacteremia cases, should also be further investigated.

Focused analyses showed that for the ST117 lineage, humans and the environment are globally a more significant source of AMR compared to animal hosts (Fig. S4A and S5). However, this difference was less pronounced when solely analyzing South American isolates from all sources (Table S3), which largely can be explained by higher carriage rates of AMR genes in animal-associated isolates from South America compared to North America, Europe, and Oceania (Fig. S4B). The result of the global analyses makes the combined interpretation with the Bayesian model predictions challenging since these putative zoonotic isolates would be expected to reflect the overall resistance observed in poultry. Perhaps for humans, these multidrug-resistant strains may be more likely to become established in the gastrointestinal tracts of people undergoing antimicrobial therapy, setting them up for subsequent multidrug-resistant extraintestinal infections, hence influencing AMR profiles in the ST117 lineage. This may be suggested by isolates in the zoonotic group carrying resistance to carbapenems (Table S4), which are not allowed in the poultry production, particularly in countries with a substantial poultry industry, and infrequently detected in avian hosts. However rare, it has also been reported and linked to contaminated feed or the farm environment (40).

South American zoonotic ST117 isolates were shown to be strongly linked as carriers of ESBLs (Fig. 4), a drug class still used in the livestock sector of this region (34). A recent study highlighted the associations between third-generation cephalosporins use in food animals and the development of ESBL in critical human pathogens (41). Furthermore, when considering not only those isolates classified as zoonotic but the whole ST117 data set, it was still evident that South American isolates from all sources were statistically more prone to carry resistance to antibiotics compared to other continents, especially antibiotics of importance in human medicine (Table S3). However, with fewer available data of African origin, and particular drug classes rarely reported in ST117 isolates in some regions (e.g., fosfomycin in North America/Europe/Oceania, or colistin in North America and Oceania, Fig. S2), this adds biases to the analyses. This should be investigated further as more human ST117 isolates become available from under-sampled regions.

### Conclusion

Food animals may be important reservoirs of zoonotic ExPEC. Prolific antimicrobial use in food animals can drive the selection of extensively antimicrobial-resistant zoonotic ExPEC lineages that can be disseminated through meat products. Here, we provide further evidence that *E. coli* ST117 is a common poultry lineage with great zoonotic potential and show that resistance to critically important antimicrobials is regionally concentrated, including in Asia and South America. Our data show that ST117 is a common cause of poultry disease outbreaks in Brazil and, owing to the public health risks of this lineage and Brazil’s role as the world’s largest poultry exporter, the implementation of further preventive disease measures is a priority.

## MATERIALS AND METHODS

### Isolate collection

Isolates were collected from outbreaks on four different Brazilian farms. Extensive mortality/morbidity occurred between 2017 and 2020 in three Brazilian poultry-producing regions (Table S1). Broiler and egg-laying farms experienced respiratory problems or signs of heavy prostration. Samples from internal organs of birds presenting characteristic colibacillosis lesions (air sacculitis, caseous on lungs, tracheal secretion, pericarditis, perihepatitis) were aseptically collected, subjected to microbiological analysis with identification of *E. coli* by MALDI-TOF MS. A total of 61 *E. coli* isolates representing individual necropsy cases among the sampled farms were randomly selected for whole-genome sequencing (WGS) (Table S1). A simplified outline of the study design is shown in Fig. S6, and a schematized map showing the location of the urban centers, poultry-producing regions, and sampled farms in Fig. S7.

### DNA extraction and whole-genome sequencing

#### Short-read sequencing

Genomic DNA was extracted on a MagNA Pure 96 automated extraction platform (Roche) using the Viral NA Small Volume DNA Multi-Sample Kit (Roche, Basel, Switzerland) according to the instructions provided by the manufacturers. Quant-iT dsDNA BR and HS Assay Kits (Thermo Fisher Scientific, Carlsbad, CA, USA) were used for DNA quantification and fluorescence was measured on a FLUOstar Omega (BMG LabTech). Sequencing libraries were prepared using the Illumina Nextera XT DNA Library Preparation Kit (Illumina, San Diego, CA, USA) and sequenced on the NextSeq 550 platform (Illumina) using a 300-cycle kit to obtain paired-end 150 bp reads.

#### Long-read sequencing

For the isolates selected for long-read sequencing, sequencing libraries were prepared with the rapid barcoding kit (SQK-RBK114.96; Oxford Nanopore Technologies, Oxford, England) according to the manufacturer’s protocol, using 200 ng DNA input. The resulting library was loaded on an R10.4.1 MinION Flowcell and sequenced *via* the MinKNOW software on a GridION sequencing instrument. Basecalling was performed with the Guppy v6.4.6 super accurate basecalling model and reads were filtered on q-score 10 with Chopper. A hybrid assembly was made with Unicycler v0.4.8 (42), using the short- and long-read reads. The circular components representing the chromosome and plasmids were extracted from the assembly graph with Bandage v.0.8.1 (43).

### Reads availability, genome assembly, and typing analyses

The raw sequencing reads of the Brazilian *E. coli* isolates are available at the European Nucleotide Archive (Bioproject PRJEB72763). Shovill v.1.0.4 (https://github.com/tseemann/shovill) was used for assembling the genomes with SPAdes (v.3.15.4). The assemblies were used for *in silico* typing with 90% identity and 80% coverage threshold settings for the detection of individual targets. The abriTAMR pipeline v.1.0.14 (https://github.com/MDU-PHL/abritamr) was used for determining virulence factors with the AMRFinderPlus v3.12.8 database, while serotype and plasmid replicons were identified with ABRicate v.0.9.0 (https://github.com/tseemann/abricate) using the EcOH and Plasmidfinder (http://genomicepidemiology.org/services/) databases, respectively. Phylogrouping was detected with EzClermont (https://ezclermont.hutton.ac.uk), ST with mlst v.2.16 (https://github.com/tseemann/mlst), and FimH with FimTyper v.1.0 (https://cge.food.dtu.dk/services/FimTyper/), and plasmid MLST (pMLST) for replicons with available database (IncF, IncI) with pmlst v.2.0. The isolates were classified as containing a ColV-like plasmid according to Liu et al. (13) using increased cutoffs (44) (≥95% identity and coverage) employing a custom BLAST database in Dotmatics’ Geneious Prime v.2022.2.2 (www.geneious.com).

### Phenotypic and genotypic AMR determination

Antimicrobial resistance (AMR) was tested with the minimum inhibitory concentration (MIC) method for each isolate using the Sensititre EUVSEC3 panel (Thermo Fisher Scientific, Massachusetts, USA). Fosfomycin MIC was determined by the agar dilution method (Liofilchem, Teramo, Italy). The clinical breakpoints values of EUCAST (45) were used, and when not available for a given antimicrobial (i.e., tetracycline, nalidixic acid), the CLSI guideline values were used (46), with *E. coli* ATCC 25922 used as control. The abriTAMR pipeline v.1.0.14 was also used for determining acquired and point-mutation antimicrobial resistance genes with the AMRFinderPLus v3.12.8 database.

### AMR index scores among the worldwide ST117 lineage

Most *E. coli* isolates obtained from the disease outbreaks among Brazilian poultry belonged to the ST117 clonal lineage (32%, *n* = 20/61, Table S1). We therefore subjected these strains to in-depth comparative analyses with publicly available genomes to investigate the zoonotic potential and the dissemination of this lineage globally.

An AMR index was established for each isolate where resistance genes corresponding to antimicrobials of critical importance to human health were assigned a higher weight in an accumulative score (32). The original index was adapted to be used on the genetic resistance markers as a proxy for the predicted phenotypic resistance. Isolates that did not contain AMR genes or AMR-related mutations were classified with a score of 0, and those positive received a weighing score which was added up to the overall sum for each isolate. Visualization of the AMR index scores as well as the combined percentage of each AMR class within countries and continents was created in R v.4.2.1 using the packages ggplot2 v.3.4.1 and rnaturalearth v.0.3.2.

### Genomic comparison analyses of Brazilian poultry isolates

The clustering of isolates was done with reference to their geographical origin and AMR profiles by a core-genome SNP-based maximum likelihood phylogeny analysis. A total of 61 *E. coli* isolates were analyzed using the NASP pipeline v.1.0.0 (47) with a whole-genome sequenced ST117 strain (GCA_004331785.1/SRP222551) as the reference. SNPs were called with GATK v.4.2.2 and recombination was removed using Gubbins (v.2.1). The phylogeny was created with IQ-TREE v.2.1.2 using ModelFinder and 100 bootstraps. Visualization was performed using iTOL v. 6.7.6 (https://itol.embl.de/).

### Phylogenetic analyses of ST117 isolates worldwide

Isolates of Brazilian origin belonging to the ST117 lineage were further analyzed in a core-genome SNP analysis as previously mentioned using the NASP pipeline with all available ST117 draft whole-genome sequences containing metadata on host and country in EnteroBase (https://enterobase.warwick.ac.uk/) (accessed on 12/2022). The genomes collected included only draft genomes of <600 contigs and with genome size between 4.6 and 5.3 Mb of isolates with available metadata regarding the source of origin (environment, animal, and human). The EnteroBase sample codes, metadata, and totals for each host source and continent of origin are listed in Table S5. Prototypic ST117 strain E44 (LXWV01000000) was used as a reference to obtain a core SNP phylogeny as previously outlined.

### Plasmid analysis

Plasmids with critical AMR gene markers were extracted using Bandage v.0.8.1 for downstream analyses. The specific contigs containing replicons carrying antimicrobial resistance genes of high priority were annotated with Bakta (v.1.7, https://github.com/oschwengers/bakta).

The plasmid core genome for each Inc type was determined using the NASP pipeline. For each Inc type, a minimum percentage threshold was considered to select a reasonable number of representative sequences (including animal, environment, and human) above a threshold similarity: For IncFII, given the number of sequences, a threshold >97% was selected; and for the lesser abundant IncI >88%, and IncX4 >70% thresholds were selected, after which GView (http://genocat.tools/tools/cgview_server.html) was used for further analyses. clinker v.0.0.27 (48) was used for visualization of genetic structures surrounding the resistance genes in relation to their comparison with complete plasmid sequences sharing a BLASTn identity >95%. Other detected replicons (listed in Table S1) were not found in contigs encoding resistance to high-priority drug classes.

### Bayesian latent class model

All the available 148 ST117 *E. coli* genomes of human origin (Table S4) were used as input for a Bayesian latent class model (17) to infer the likelihood of those having animal origin based on host-specific MGEs. The model generated a host-origin probability score for each isolate by inferring latent classes of human or meat using multivariate binary responses from the whole set of MGEs, along with clade information from the pan-ST core genome phylogeny. Response probabilities were generated using independent logistic normal priors with mean 0 and standard deviations of 1.5 while class probabilities use a Beta (1, 1) prior.

### Statistics and data visualization

Statistical tests were made in R v.4.2.1. Univariate logistic regression analyses were performed to determine whether there was an overrepresentation of resistance to any drug classes in ST117 collected from South American isolates compared to isolates from other continents. Also, logistic regression analysis was used to test whether ST117 isolates of animal origin more often carried resistance genes compared to isolates collected from other sources (i.e., human or environment). These tests were made for all isolates of the global collection, as well as on a subset only including South American isolates. The univariate logistic regression analyses were made for each of the resistance genes that were present in a minimum of 5% and no more than 95% of the isolates across the entire ST117 collection. *P*-values were corrected for multiple testing using the Benjamini and Hochberg method, where adjusted *P*-values < 0.05 were considered to be of significance.

A principal component analysis (PCA) of the genetic-based AMR profiles of FZEC ST117 isolates was conducted to examine whether AMR profiles differed according to the continent of origin. The PCA was created using the R packages factoextra v.1.0.7 and FactoMineR v.2.6 using a presence/absence (1/0) matrix with information on genetic-based resistance to 14 antibiotics as the input.

## Data Availability

The sequencing data from this study was uploaded to the European Nucleotide Archive (ENA), BioProject number: PRJEB72763.

## SUPPLEMENTAL MATERIAL

Supplemental material spectrum.00466-24-s0001.pdf Fig. S1 to S7.

Table S1 spectrum.00466-24-s0002.xlsx Extended details on the genotypic and phenotypic results of the 61 APEC isolates analyzed in this study.

Table S2 spectrum.00466-24-s0003.xlsx Plasmid details of the six long-read sequenced ST117 APEC isolates of this study, featuring farm and isolate origin, virulence and AMR carriage, and highest identity replicons.

Table S3 spectrum.00466-24-s0004.xlsx Univariate logistic regression analysis indicating significant AMR overrepresentation in ST117 isolates from South America compared to other continents.

Table S4 spectrum.00466-24-s0005.xlsx Details on the 148 ST117 isolates of human origin tested with the Bayesian latent class model inferring the likelihood of an animal origin based on host-specific mobile genetic elements.

Table S5 spectrum.00466-24-s0006.xlsx Accession number and available metadata for the 1,719 worldwide ST117 isolates analyzed in this study.

ASM does not own the copyrights to Supplemental Material that may be linked to, or accessed through, an article. The authors have granted ASM a non-exclusive, world-wide license to publish the Supplemental Material files. Please contact the corresponding author directly for reuse.
