# Supplementary material for: Escherichia coli ST117: exploring the zoonotic hypothesis
Source: Microbiol Spectr. 2024 Sep 6;12(10):e00466-24. doi: 10.1128/spectrum.00466-24 (PMC11448156; doi:10.1128/spectrum.00466-24)
Supplement: Supplemental material — Fig. S1 to S7. [file spectrum.00466-24-s0001.pdf]

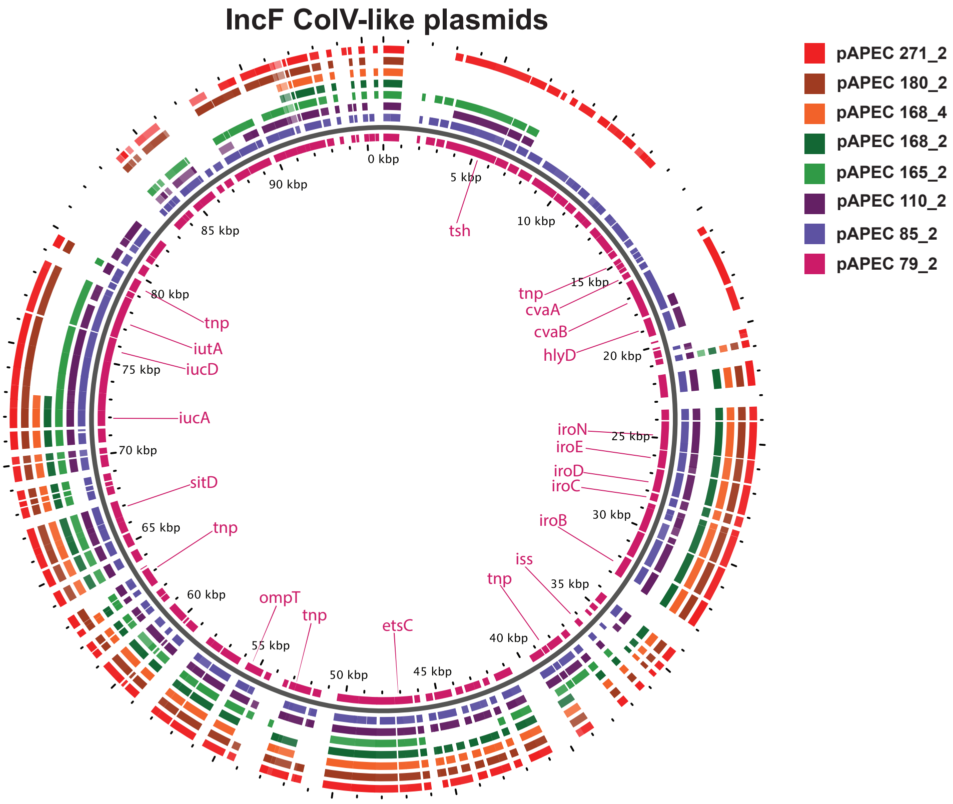

Suppl. Figure 1: Circular visualization of the IncF ColV-like plasmids identified in the long-read sequenced isolates indicating most of the virulence factors common to all isolates.

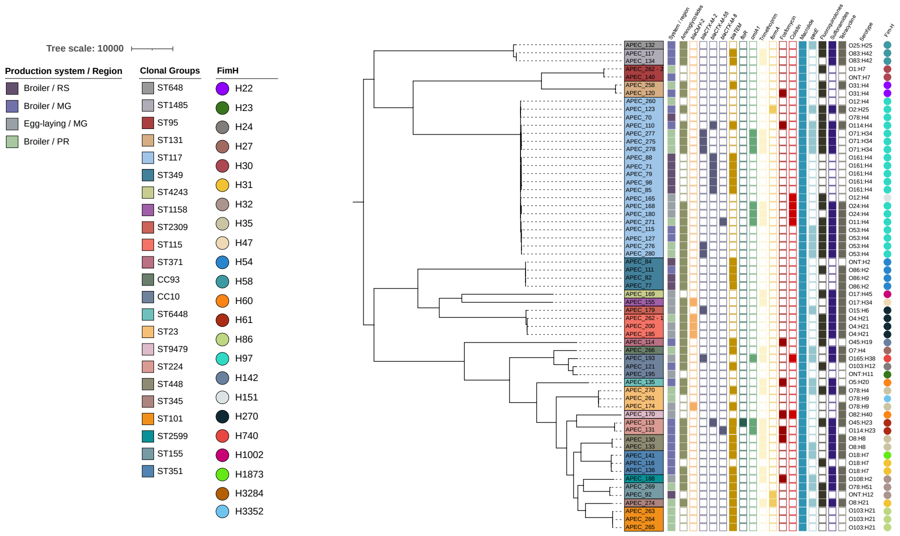

Suppl. Figure 2: Core genome phylogeny of the 61 APEC isolates of this study containing metadata for each isolate regarding the farm system and region, MLST, and detected AMR gene/class. The tree is midpoint rooted. The phylogeny was based on SNP calling in 58% (3,78Mb) of the reference genome, and the scale bar indicates substitutions per site.

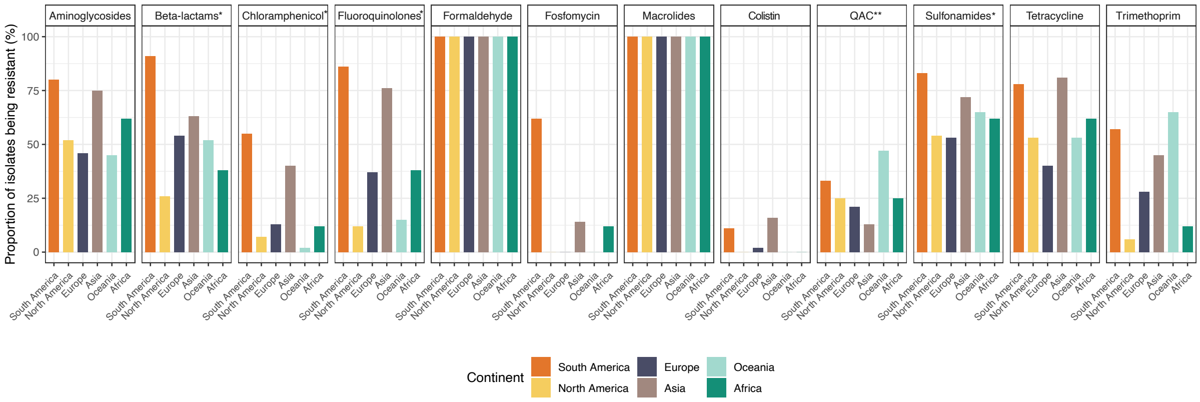

Suppl. Figure 3: Bar chart depicting the percentage of each antimicrobial class detected in the ST117 lineage of worldwide isolates according to each continent of origin. *indicates antibiotic classes significantly more present in South American isolates compared to all continents; **indicates quaternary-ammonium.

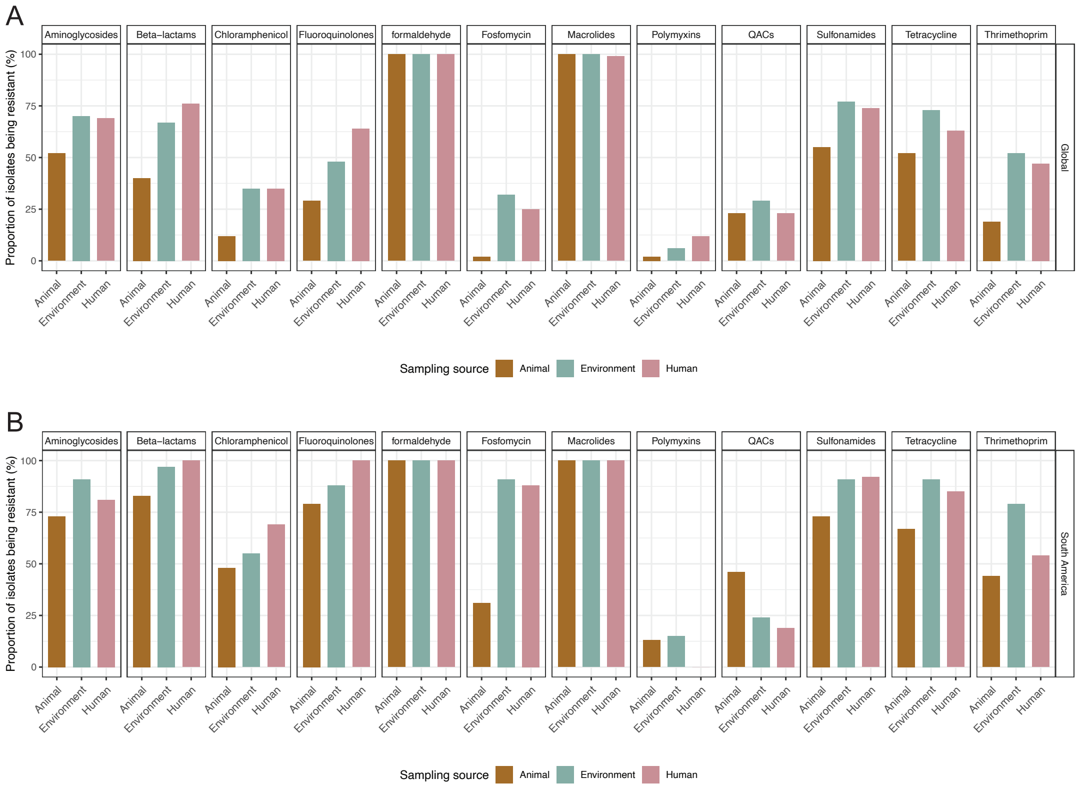

Suppl. Figure 4. Bar charts depicting the percentage of each antimicrobial class detected in the ST117 lineage: A: In worldwide ST117 isolates according to each host. B: In South American ST117 isolates according to each host.

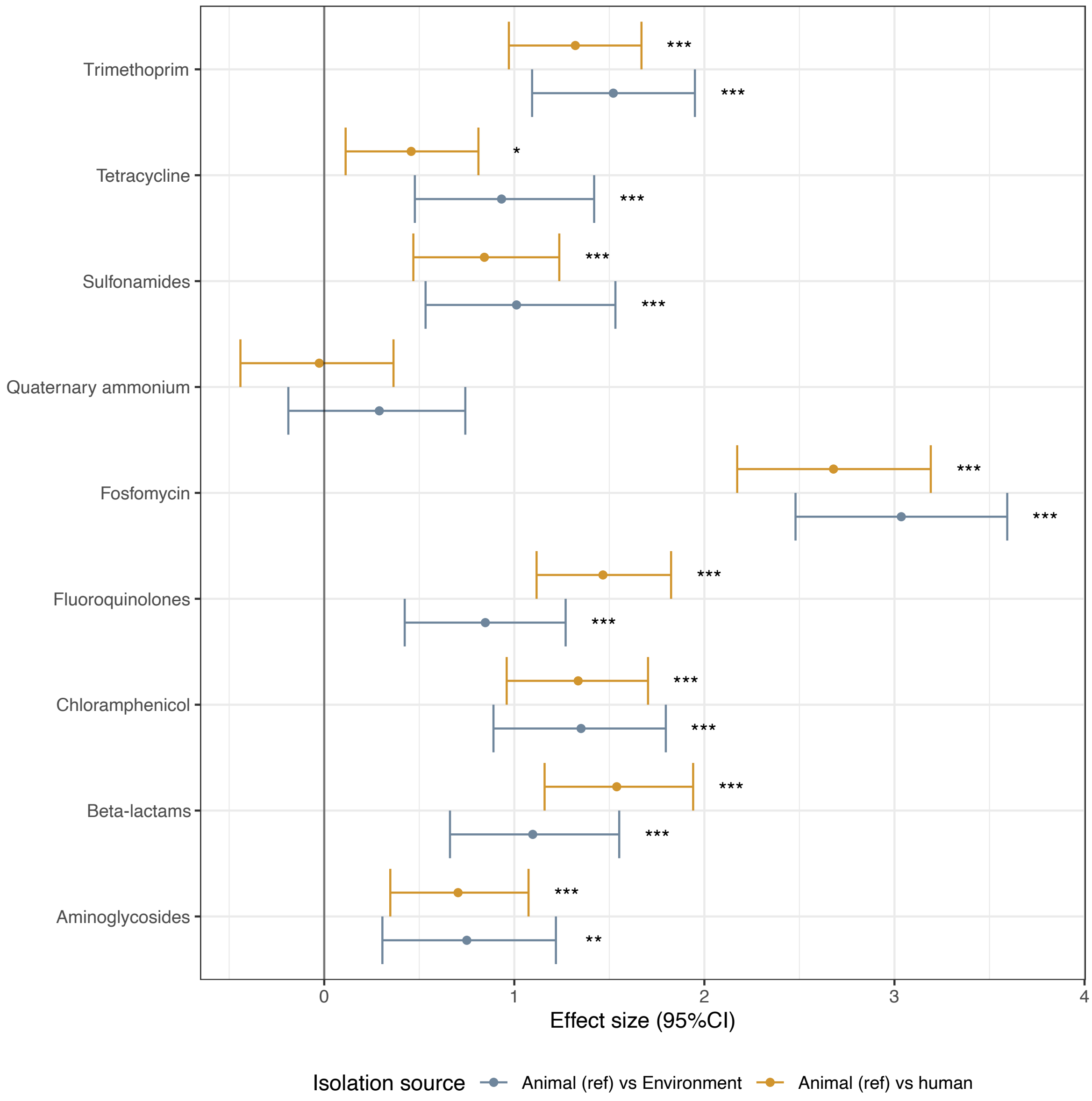

Suppl. Figure 5: Univariate regression analysis plot of the AMR detected in worldwide ST117 isolates comparing the animal source to human and environmental sources, respectively. Log-odds (dots) are shown with 95%CI (error bars). Stars indicates significance based on the false discovery rate (FDR) adjusted p-values (***<0.001, **<0.01, *<0.05). An estimate above 0 shows greater chance of having the specified AMR genes within the test group compared to the reference group (Animal).

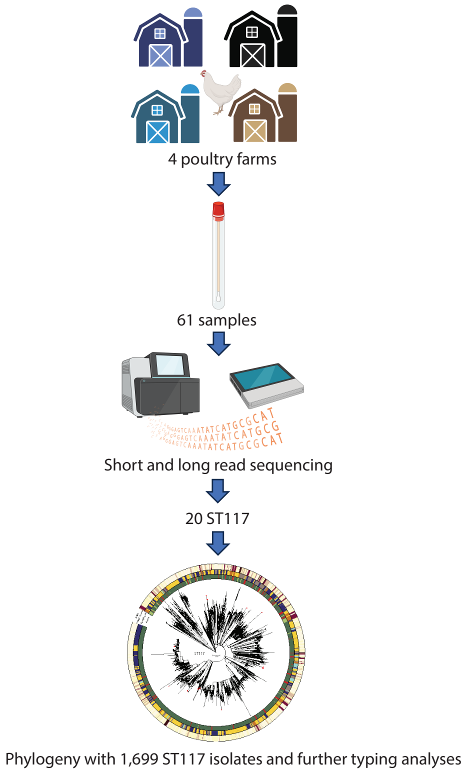

Suppl. Figure 6: Study design overview for the APEC isolates of this study and downstream analyses**.**

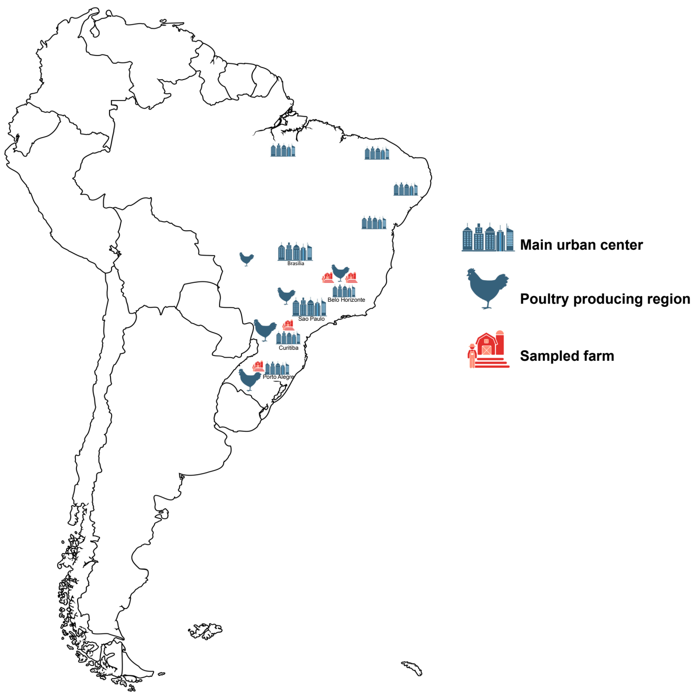

Suppl. Figure 7: Schematized map showing the location of the main urban centers, poultry producing regions, and the sampled farms. The relative size indicates larger/important urban centers/poultry production regions.
